# Functional Stability of the Human Kappa Opioid Receptor Reconstituted in Nanodiscs Revealed by a Time-Resolved Scintillation Proximity Assay

**DOI:** 10.1371/journal.pone.0150658

**Published:** 2016-04-01

**Authors:** Randi Westh Hansen, Xiaole Wang, Agnieszka Golab, Olivier Bornert, Christine Oswald, Renaud Wagner, Karen Laurence Martinez

**Affiliations:** 1 Department of Chemistry & Nano-Science Center, University of Copenhagen, Copenhagen, Denmark; 2 CNRS UMR7242/Laboratoire d’excellence MEDALIS, Institut de Recherche de l’ESBS, Biotechnologie et Signalisation Cellulaire, Université de Strasbourg, Illkirch, France; 3 Department of Neuroscience and Pharmacology, University of Copenhagen, Copenhagen, Denmark; University of Connecticut, UNITED STATES

## Abstract

Long-term functional stability of isolated membrane proteins is crucial for many *in vitro* applications used to elucidate molecular mechanisms, and used for drug screening platforms in modern pharmaceutical industry. Compared to soluble proteins, the understanding at the molecular level of membrane proteins remains a challenge. This is partly due to the difficulty to isolate and simultaneously maintain their structural and functional stability, because of their hydrophobic nature. Here we show, how scintillation proximity assay can be used to analyze time-resolved high-affinity ligand binding to membrane proteins solubilized in various environments. The assay was used to establish conditions that preserved the biological function of isolated human kappa opioid receptor. In detergent solution the receptor lost high-affinity ligand binding to a radiolabelled ligand within minutes at room temperature. After reconstitution in Nanodiscs made of phospholipid bilayer the half-life of high-affinity ligand binding to the majority of receptors increased 70-fold compared to detergent solubilized receptors—a level of stability that is appropriate for further downstream applications. Time-resolved scintillation proximity assay has the potential to screen numerous conditions in parallel to obtain high levels of stable and active membrane proteins, which are intrinsically unstable in detergent solution, and with minimum material consumption.

## Introduction

Membrane proteins (MPs) are key components in cell communication. They act as transporters of molecules across the plasma membrane or they function as enzymes or receptors that transmit outside stimuli to intracellular responses. Because of their predominant role in cellular signaling and physiological processes, altered function of MPs are involved in many severe diseases. Consequently MPs are targets for more than 60% of approved drugs on the market [1].

In combination with cell-based screening approaches, an important stage in early drug discovery processes, and rational drug design is often experimental verification and validation on proteins isolated from the complex cellular environment [2–4]. During this process, a combination of structural studies, together with biochemical, and biophysical studies is essential for lead identification and optimization to develop potential new drug candidates [5]. For this purpose, a wide range of complementary methods and techniques are used, such as biochemical analyses to elucidate the pharmacology of identified new hits [6,7], and biophysical methods, including fluorescence spectroscopy [8], solid state nuclear magnetic resonance (NMR) [4,9,10], and surface plasmon resonance (SPR) [11,12] to determine the protein structure and relate it to thermodynamic and kinetic binding parameters of ligand-protein interactions.

For the above-mentioned methods, there are several requirements regarding the sample quality of the isolated proteins. In the case of MPs, this is a tremendous challenge because of their hydrophobic core. MPs have to be produced at high quantities, extracted from cell membranes, and are often purified in detergent micelles. Although mild detergents are used to handle MPs in solution, detergents often have a dramatic effect on the biological function, especially if the proteins are exposed to detergents for extended periods [13,14]. Despite the development of new strategies and techniques to stabilize MPs in the last two decades, it is still an extensive challenge to isolate functional and stable MPs, especially integral MPs such as G protein coupled receptors (GPCRs) [15–18].

One successful approach to stabilize the biological activity of MPs, is to generate thermostabilized proteins suitable for e.g. small compound screening by SPR and NMR [19]. This approach has been successfully adapted to several GPCRs [20,21]. However, this method is often laborious due to the protracted process of generating thermostabilized GPCRs that still have their original biological properties retained after genetic modifications. A different approach is to stabilize native GPCRs in a lipid environment, such as lipoprotein particles known as Nanodiscs or the similar high-density lipoprotein (rHDL) particles. The lipoprotein particles consist of a lipid bilayer encircled by two amphipathic membrane scaffold proteins (MSPs). The surrounding MSPs maintain the lipid bilayer as well-defined nanometer-sized particles. When lipids, MSPs and target MPs are mixed in detergent solution, MPs are reconstituted in Nanodiscs by self-assembly upon detergent removal. Thus the lipoprotein particles can be handled as soluble proteins, while the MPs can reside in a physiologically relevant medium. Nanodiscs and rHDL have proven to be a powerful method to stabilize and handle MPs in aqueous solutions. Numerous MPs, with a broad range of biological functions, have been reconstituted in the lipoprotein particles [15]. Furthermore, the above-mentioned biophysical methods have been shown to be compatible with MPs/Nanodisc complexes [9,11,22–24]. However, there is insufficient evidence of the functional stability of MPs in Nanodiscs, for periods compatible with biophysical and pharmacological investigations.

In order to quantify the functional stability of a GPCR reconstituted in Nanodiscs, we have extended a well-established radioactive assay, scintillation proximity assay (SPA), to measure time-dependent high-affinity ligand binding to a GPCR. SPA is a radioligand surface sensitive technique, based on beads filled with scintillant. This allows specific detection of receptor bound ligands without prior separation from free ligand. Furthermore, the technique reduces material consumption and allows higher throughput, compared to conventional filter-based methods [6]. SPA has been utilized for a broad class of drug targets, and the assay can be used for MPs in various environments, including membrane fractions, detergent solution [6,25,26] or reconstituted in Nanodiscs [27,28].

To evaluate SPA as a time-resolved stability assay, we measured high-affinity ligand binding to a representative GPCR, the kappa opioid receptor (KOR), either in detergent solution or reconstituted in Nanodiscs. KOR is a class A GPCR widely distributed in the nervous system, where it is involved in the regulation of behavioral processes as perception, reward and mood [29]. The receptor responds to the endogenous peptide ligand dynorphin A, which regulates various functional pathways in the brain. As a consequence, KOR is believed to have a crucial role in neuropsychiatric disorders such as depression, stress and addiction and is hence a pharmaceutical important drug target [30]. The crystal structure of KOR has been solved recently, nevertheless biochemical and biophysical investigations on the isolated receptor are still limited due to the poor stability of the protein in detergent [31].

Using time-resolved SPA, we show that the ligand binding activity of KOR decreases rapidly when KOR is in detergent micelles. When KOR is reconstituted into Nanodiscs the receptor pharmacology is restored and most importantly the ligand binding activity is stable over several hours at room temperature. We demonstrate that SPA can be used as a quick and easy approach using minute amounts of receptor, to quantify the stability of GPCRs required for many *in vitro* applications. Time-resolved SPA can easily be implemented in routine and automated drug discovery procedures to screen conditions for long-term stability of isolated MPs in general.

## Materials and Methods

### Expression of KOR in *Pichia pastoris* and isolation of membrane

Human kappa opioid receptor (KOR) was expressed in *Pichia pastoris* (*P*. *pastoris*) as previously described [32]. The KOR sequence contained a N-terminal Flag-tag and a decahistidine-tag followed by a tobacco etch virus (TEV) protease site. After the KOR reading frame the C-terminus comprised a second TEV site followed by the biotinylation domain of the transcarboxylase from *Propionibacterium shermanii*. Yeast cells expressing KOR, were broken with glass beads (Sigma beads, acid washed, G8772-1kg, 0,5 mm in diameter) using a BeadBeater (Biospec Products). All procedures were performed at 4°C or on ice. Cells were suspended in lysis buffer (50 mM Tris-HCl pH 7.4, 0.5 M NaCl, 0.1 mM TCEP, 1 mM PMSF, 5% glycerol, 5 mM EDTA, 1 tablet of Complete protease inhibitor cocktail). Membranes were separated from cell debris by low-speed centrifugation of 3000 x g for 5 min at 4°C. The retained supernatant was further centrifuged at 120000 x g for 30 min at 4°C (Sorvall discovery M120, Hitachi) to collect the membranes. The membranes were re-suspended in membrane buffer (50 mM Tris-HCl pH 7.4, 0.5 M NaCl, 0.1 mM TCEP, 1 mM PMSF, 5% glycerol, 20 μM naltrexone (Sigma)). Protein concentration was quantified by bicinchoninic acid (BCA) assay (Pierce) using bovine serum albumin as standard and following the manufacture’s instructions. Prepared membranes were stored at -80°C.

### Radioligand filter binding assay

Specific ligand binding to KOR in *P*. *pastoris* membranes was measured by radioligand filter binding assay. Isolated *P*. *pastoris* membranes were diluted in binding buffer (50 mM Tris-HCl pH 7.4, 0.5 M NaCl, 1mg/ml BSA) and mixed in triplicates with radioligand [^3^H]-diprenorpine (DPN, 42.3 Ci/mmol, Perkin Elmer) in concentrations varying from 0.08 to 10 nM in a 96 well plate (MultiScreen HTS-FC 1.2/0.65 μm glass fiber filter), which was pre-wetted in washing buffer (50 mM Tris-HCl buffer, pH 7.4). Non-specific binding was measured in parallel by including 50 μM naloxone. For each binding experiment, membranes corresponding to 3–5 μg/well were used keeping the ratio of bound radioligand <10% of the total added radioligand. The assay mixture was incubated for 2 h at room temperature. To separate bound from free ligands, the plate was rapidly filtrated and washed four times with 100 μl ice-cold washing buffer. Scintillation liquid (Ultima Gold XR, Perkin Elmer) was added to the wells and the assay was counted by a liquid scintillation counter (Wallac Microbeta TriLux 1450). Specific binding was calculated and *K*_*D*_ was measured from saturation curves fitted to a one-site binding model using Igor Pro 6.1.

### Extraction of membrane proteins in detergent

All steps were done at 4°C or on ice. Isolated membranes were diluted to 5 mg/ml with 50 mM Tris-HCl, pH 7.4, 500 mM NaCl, 5% glycerol, 1 mM PMSF, 0.1 mM TCEP, 10 mM imidazole. When solubilized MPs were used for purification, 20 μM NTX was included in the buffer. Detergent n-Dodecyl-β-D-maltoside (DDM) mixed with cholesteryl hemisuccinate tris salt (CHS) was added to a final concentration of 1%/0.2% DDM/CHS (w/v) and incubated for 15 min at 4°C. The suspension was centrifuged for 20 min at 120000 x g to pellet non-solubilized material. Solubilized MPs were used directly for SPA assay or purification.

### Purification and reconstitution in Nanodiscs

Solubilized MPs were purified by immobilized metal ion affinity chromatography (IMAC) on a HisTrap HP column (GE Healthcare, 1 ml) connected to a manual pump at 4°C. The column was equilibrated in purification buffer (50 mM Tris-HCl, 500 mM NaCl, 5% glycerol, 1 mM PMSF, 0.1 mM TCEP, 20 μM NTX with 10 mM imidazole, 0.1%/0.02% DDM/CHS (w/v), pH 7.4). Solubilized proteins were loaded on the column and washed in purification buffer until baseline was reached (UV absorbance 280 nm). Proteins were eluted by elution buffer (50 mM Tris-HCl, 500 mM NaCl, 5% glycerol, 1 mM PMSF, 0.1 mM TCEP, 500 mM imidazole, 20 μM NTX, 0.1%/0.02% DDM/CHS (w/v), pH 7.4) and eluted fractions containing protein were pooled. The membrane scaffold protein MSP1E3D1 construct was provided by the group of S. Sligar. Expression and purification was carried out as previously published [33]. POPC (1-palmitoyl-2-oleoyl-*sn*-glycero-3-phosphocholine) and POPG (1-palmitoyl-2-oleoyl-*sn*-glycero-3-phospho-(1'-*rac*-glycerol)) lipids (Avanti Polar lipids) were prepared by dissolving lipids (3:2 molar ratio POPC:POPG) in chloroform and dried with a gentle stream of nitrogen on the edge of a glass tube. MSP1E3D1 and POPC:POPG were mixed in 20 mM Tris-HCl pH 7.4, 100 mM NaCl, 50 mM sodium cholate (final concentration) buffer in the ratio 1:110 MSP:lipids (molar ratio). IMAC purified proteins were mixed immediately after elution from the HisTrap HP column with lipids (POPC:POPG 3:2, molar ratio) and MSP1E3D1 in a ratio of 1:10, protein:MSP (molar ratio) and incubated 15 min on ice. Self-assembly of Nanodiscs was initiated by detergent removal with the addition of BioBeads SM-2 (Biorad) 1 mg/ml. Samples incubated with biobeads overnight at 4°C and were then removed by filtration (0.22 μm). KOR/Nanodisc complexes were further purified utilizing an anti-Flag M2 Affinity Gel (Sigma). The column was prepared according to the manufactures recommendations. 1 ml resin was equilibrated in standard buffer (20 mM Tris-HCl pH 7.4, 100 mM NaCl), sample was mixed with the resin and incubated for 2 h at 4°C. After incubation, the column was washed in standard buffer until UV absorbance 280 nm reached a value below 0.01 AU. Anti-Flag peptide was diluted in standard buffer to 0.15 mg/ml, mixed with the resin and the mixture was incubated at 4°C for 30 min. Subsequently, KOR/Nanodisc complexes were eluted from the column by washing the column in standard buffer. Fractions containing protein (UV absorbance 280 nm) were collected and concentrated (30 kDa MWCO, MilliPore). Finally, the concentrated protein was injected onto a Superdex 200 10/300 GL (GE Healthcare) pre-equilibrated with standard buffer on an ÄKTA FPLC system at a flow rate of 0.5 ml/min. To estimate the Stokes radius of the eluted proteins, the Superdex 200 column was calibrated with standard proteins from gel filtration calibration kits LMW and HMW (GE Healthcare). Eluted proteins, corresponding to KOR/Nanodisc complexes, were collected and concentrated (30000 Da MWCO, MilliPore) to a protein concentration corresponding to approximately 0.2 mg/ml. Samples were either stored at 4°C or frozen and stored at– 80°C.

### SDS-PAGE and Western blotting

KOR/Nanodisc samples were diluted in NuPage LDS buffer including 0.3% SDS and separated on a 10% Bis-Tris gel (NuPAGE, Invitrogen) in MES buffer at 200 V. The gel was either stained in Coomassie blue (Sigma) or transferred to a methanol pre-treated PVDF membrane (Invitrogen) for 1 hour at 30 V in transfer buffer (10 mM Tris-HCl, 150 mM NaCl, 0,05% Tween-20, pH 7.5). The PVDF membrane was washed in ultra pure water and blocked in blocking buffer (10 mM Tris-HCl, 150 mM NaCl, 0.05% Tween-20, 3% milk powder BioRad) for 1 hour at room temperature. KOR was detected through the C-terminal biotin-tag by incubation with streptavidin alkaline phosphatase (Sigma), diluted (1:10000) in blocking buffer, and incubated for 1 hour with the PVDF membrane. The blot was washed 3 times in washing buffer (10 mM Tris-HCl pH 7.5, 150 mM NaCl, 0.05% Tween-20) and finally, the colorimetric reaction was developed for 5–30 min with alkaline phosphatase substrate, NBT/BCIP (Thermo Scientific). The intensity of bands of the Coomassie blue stained gel was analyzed by ImageJ Gel Analyzer.

### MALDI mass spectrometry

From the Coomassie blue stained gel the band corresponding to KOR was cut and analyzed on a Bruker Autoflex Speed MALDI TOF/TOF instrument. Before loading, protein samples were reduced and alkylated with iodoacetamide and subsequently digested with trypsin. The resulting peptides were concentrated and eluted onto an anchorchip. The peptide mixture was analyzed in positive reflector mode. The MS and MS/MS spectra were combined and used for database searching using the Mascot software.

### SPA assay

All procedures were performed on ice or at 4°C until assay counting was initiated at room temperature (23°C). Time-resolved stability assay with detergent-extracted MPs was carried out directly after solubilization in binding buffer I (20 mM Tris-HCl, pH 7.4, 500 mM NaCl, 5% glycerol, 1 mM PMSF, 0.1 mM TCEP, 0.4% BSA). Solubilized proteins were diluted in the assay resulting in a final concentration of 0.1% DDM in the assay buffer. Stability assays performed with KOR reconstituted in Nanodiscs were done in binding buffer II (20 mM Tris-HCl pH 7.4, 100 mM NaCl pH 7.4, 0.4% BSA). KOR/Nanodisc samples were added at quantities corresponding to approximately 30 fmol/well. Samples were mixed with 0.5 mg/well yttrium silicate (YSi) streptavidin coated SPA beads (Perkin Elmer) in binding buffer and a final concentrations of 10 nM [^3^H]-DPN (42.3 Ci/mmol, Perkin Elmer) in a total volume of 200 μl. In parallel, the non-specific binding was measured including 50 μM naloxone in the assay. All measurements were performed in triplicates in a white-wall clear-bottom 96-well plate (Wallac). The assay was gently shaken on a vibrating platform at 4°C for 1.5 hours to equilibrate ligand binding and immobilization of receptors on the SPA beads. After incubation the assay was counted with a liquid scintillation counter (Wallac Microbeta TriLux 1450, SPA cpm mode) repeatedly with 17 min between measurements at room temperature. As a control, the same assay conditions as described above were performed at RT and bound and free radioligands were separated at specific time points on Sephadex G-50 gravity-flow columns (GE Healthcare) equilibrated in binding buffer. After applying 50 μl of samples, columns were centrifuged for 2 min at 1000 x g. Separated samples were mixed with scintillation liquid and incubated for 1 hour at RT, before counting on a liquid scintillation counter. As data analysis, specific binding was normalized to the initial binding at time zero, corresponding to initiation of assay counting at RT. The half-life of high-affinity ligand binding was calculated from fitting to single or double exponential decay functions in Igor Pro 6.1.

In SPA saturation binding experiments, KOR/Nanodisc (30 fmol) complexes were mixed as above but with various concentrations of [^3^H]-DPN (0.08–10 nM). In parallel non-specific binding was measured in the presence of 50 μM naloxone. During purification and reconstitution active KOR was up-concentrated, as compared to the detergent solubilized receptor sample. Therefore, saturation and inhibition binding assays were performed with reduced SPA beads/well; 0.25 mg/well of streptavidin-coated SPA YSI beads. Competition binding assays were performed with similar conditions, but with various concentrations of either naloxone or dynorphin A (1–17) (Anaspec) corresponding to final ligand concentrations of 10^−11^ to 10^−4^ M. To measure *K*_*D*_ and *IC50*, data corresponding to specific binding, were fitted to a one-site binding model using Igor Pro 6.1. *Ki* was measured from the *IC50* value.

### G-protein binding assay

Heterotrimeric G_i_ proteins (Gα_i_ bovine, His6-rat Gβ_1_ and bovine Gγ_2_) were expressed in *Sf9* insect cells and co-purified as heterotrimeric proteins by IMAC chromatography as described in [34]. GTPγS binding experiments were carried out using the fluorescent BODIPY FL GTPγS (Life Technologies) analog as described in [35]. Binding reactions were done in assay buffer 50 mM Tris-HCl, pH 7.4, 150 mM NaCl, 100 μM EDTA, 3 mM MgCl_2_. G_i_ proteins were first diluted in assay buffer including 0.012% DDM and were further diluted in the assay having a final concentration of 0.0012% DDM. KOR/Nanodisc complexes, G_i_ proteins and BODIPY FL GTPγS were mixed having final concentrations of 10 nM active receptors, 100 nM G_i_ proteins and 100 nM BODIPY FL GTPγS. The assay was incubated for 25 min at 20°C and fluorescence emission was recorded at 509 nm (± 3 nm) with an excitation at 490 nm (±2 nm) on a FluoroMax-4 Spectrometer. In presence of ligand, KOR/Nanodisc complexes were pre-incubated with 1 μM dynorphin A (1–17) (Anaspec). As a control, the same assay was performed with similar concentration of empty Nanodiscs. All data were corrected for BODIPY FL GTPγS background.

## Results

KOR was expressed in *P*. *pastoris* with a N-terminal Flag- and decahistdine-tag, and a C-terminal biotin-tag as earlier described [32]. Receptors were extracted from isolated membrane fractions in a mixture of n-Dodecyl-β-D-maltoside (DDM) and the cholesterol-like molecule cholesteryl hemisuccinate (CHS) to improve the stability of KOR (1%/0.2% DDM/CHS (w/v)) [36]. The time dependent stability of high-affinity ligand binding to KOR in detergent micelles was assayed by time-resolved SPA.

### Ligand binding stability to detergent solubilized KOR measured by time-resolved scintillation proximity assay

**Fig 1:**
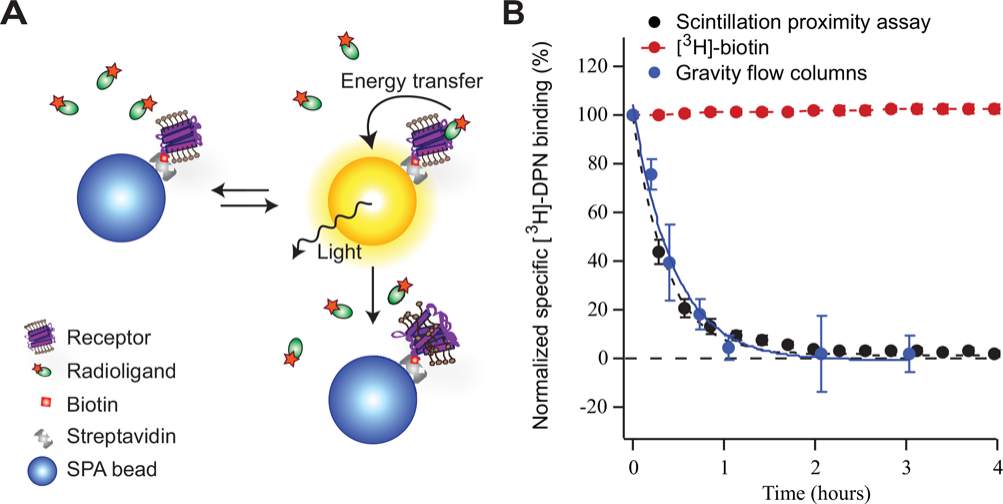
Principle of time-resolved Scintillation Proximity Assay (SPA) to quantify stability of high-affinity radio-ligand binding to GPCRs. A) Receptors are immobilized on the SPA beads. When radioligands bind to immobilized receptors, energy from the radioisotope decay is efficiently transferred to the scintillator contained in the beads, thus generating a detectable light signal. If the buffer, including detergent, causes the loss of receptor affinity to radiolabeled ligand, the signal will start to decrease irreversibly. The decay of high-affinity ligand binding to receptors can be measured when the same assay is counted repeatedly over time. B) Comparison of desalting gravity flow separation columns (blue circles) and SPA (black circles) to measure decrease in binding of [^3^H]-DPN to KOR over time at room temperature. KOR was solubilized in 1%/0.2% DDM/CHS (w/v) and diluted in the assay to 0.1%/0.02% DDM/CHS (w/v). Specific binding was normalized to initial binding. As control of assay stability, the signal of bead-immobilized [^3^H]-biotin was measured in parallel (red circles). Error bars are standard error of the mean from four (gravity flow columns) or three (SPA) independent experiments each done in triplicates. To quantify the stability of ligand binding, data was fitted to an exponential decay function (solid and dashed lines).

SPA is a homogeneous assay technology, where the target protein is immobilized on beads filled with a scintillation matrix. Only radioligand within close proximity to the SPA beads can efficiently transfer energy from the radioisotope decay to the scintillator inside the beads, which generates a light signal (Fig 1) [6,37,38]. Thereby, it is not required to include a filtration step to separate bound from free ligand in a receptor-binding assay. Monitoring the same assay over longer period of time, allows the detection of changes in ligand binding, such as reduction in ligand binding to the receptor, due to loss of receptor stability (Fig 1A).

Detergent solubilized KOR was immobilized on streptavidin coated SPA beads utilizing the C-terminal biotin-tag. The samples were equilibrated at 4°C, with radiolabeled antagonist, [^3^H]-DPN, at a concentration corresponding to saturating conditions for high-affinity binding (10 nM [^3^H]-DPN ≈ 10 x *K*_*D*_). The time-dependent ligand binding to KOR was measured by counting the assay repeatedly for several hours at room temperature (23°C). As seen on Fig 1B, the specific binding of [^3^H]-DPN to KOR decreased rapidly and with a complete loss of ligand binding activity within 100 minutes at room temperature (Fig 1B).

To confirm that the observed decay was a result of KOR loosing its ligand binding capacity, we used a conventional ligand binding assay based on desalting gravity flow columns, to measure specific ligand binding to detergent solubilized receptors incubated at room temperature at various time points [39,40]. Using the same assay conditions, samples were applied to the gravity flow columns to separate bound from free ligand. The measured decrease in ligand binding signal detected by the two different assay techniques were similar. Their fits by an exponential decay function [41] (Fig 1B) provided similar half-life of high-affinity ligand binding to receptors: 15.3 ± 0.5 minutes and 18.5 ± 2.3 minutes measured by SPA and by gravity flow columns, respectively. However, the higher number of data points obtained with the SPA method allowed a more detailed measurement of the ligand binding activity decay and better fit of the data, than the measurements done with desalting gravity flow columns.

Comparison of the data from the two different assay methods, demonstrated that the observed decay of ligand binding is in fact due to receptors losing their high-affinity for the ligand and not as consequence of immobilization on the SPA beads. Furthermore, the assay stability was confirmed by the continuous signal from [^3^H]-biotin immobilized on the streptavidin coated SPA beads.

Compared to other separation techniques, such as desalting gravity flow columns, many experimental conditions could be handled in parallel, which increases the throughput and allows for rapid optimization of conditions that stabilize the function of MPs in detergent. In addition, a large number of data points are obtained in one assay and the consumption of materials is reduced considerably, since the same assay plate is counted repeatedly. This makes SPA an efficient method to screen for optimal solubilization conditions that stabilize the biological function of MPs.

We tested several detergents and solubilization conditions, using SPA as a screening method. Only minor changes in the measured stability of ligand binding to KOR were observed (S1 Fig). In all cases, detergent had a dramatic effect on the ligand binding ability of KOR. The rapid degradation of the high-affinity ligand binding to KOR, made it essential to stabilize the receptor in an environment different from detergent micelles, to maintain a fully functional receptor over time that was suitable for downstream analysis.

### Purification and reconstitution in Nanodiscs

To stabilize KOR in a functional state for extended periods of time, we aimed at reconstituting the receptor in Nanodiscs as a defined lipid bilayer. Two main approaches have been used to reconstitute MPs in Nanodiscs. One is to purify MPs using multiple chromatography steps prior to reconstitution in Nanodiscs [39,42]. It requires that MPs remain functional during purification or that their function is restored after reconstitution. Another strategy, used for very unstable MPs, is to capture all extracted MPs in Nanodiscs right after solubilization and purify the target protein after stabilization in Nanodiscs [10,43–45]. Using this later approach minimizes the time MPs are in contact with detergent micelles. However, compared to purified proteins, a larger amount of material is reconstituted, thus the method may require vast amounts of lipids and MSP. We chose an intermediate approach, where solubilized KOR was quickly purified by IMAC, followed by reconstitution into Nanodiscs and several subsequent purification steps.

**Fig 2:**
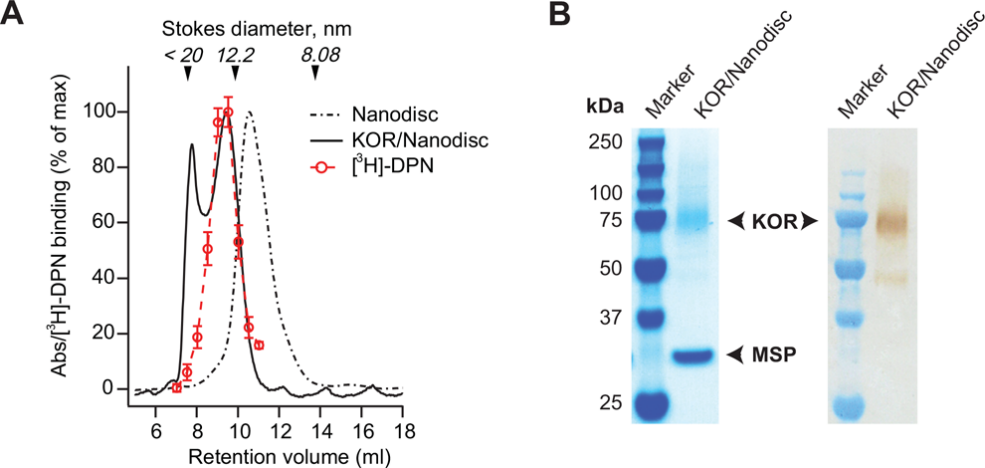
Reconstitution of KOR in Nanodiscs. A) SEC chromatogram (Superdex200) representing UV_280 nm_ traces from resolution of empty Nanodiscs (black dotted line) and of KOR/Nanodisc complexes (black line). KOR/Nanodisc sample eluted as two peaks. The second peak on the chromatogram corresponded to Nanodiscs containing active KOR, confirmed by specific binding of [^3^H]-DPN (10 nM) to KOR in eluted fractions (red circles). The column was calibrated with standard proteins indicated by arrows. The Stokes diameters of empty Nanodiscs and KOR/Nanodisc complexes were calculated to be 11.9 nm and 14.1 nm respectively. B) SDS-PAGE detection of KOR reconstituted in Nanodiscs. Left: Coomassie brilliant blue staining, KOR (66 kDa) was visible around ≈ 75 kDa while the MSP1E3D1 protein (32.6 kDa) was visible at ≈ 30 kDa. Right: The presence of KOR was confirmed by western blot detection with streptavidin alkaline phosphatase binding to the C-terminal biotin-tag of KOR. Original uncropped and unadjusted gel and blot can be seen in S2 Fig.

The first IMAC purification removed a large part of other solubilized MPs and reduced detergent concentration from 1% to 0.1% DDM, which facilitated detergent removal and the initiation of Nanodiscs self-assembly. To favor the assembly of a single receptor per Nanodisc, reconstitution was prepared with a 10-fold molar excess of MSP1E3D1 to IMAC purified KOR, and with a lipid composition consisting of POPC and POPG (3:2). The 10-fold ratio has shown to be optimal for the assembly of one GPCR per Nanodisc [46,47], and this lipid composition has been found to be optimal for other GPCRs [39,48,49]. After reconstitution and detergent removal, KOR was further purified by its N-terminal FLAG-tag using affinity chromatography (anti-FLAG M2). In addition to increase sample purity, the anti-FLAG purification step removed excess of empty Nanodiscs. As a last separation step, KOR/Nanodisc complexes were separated by size exclusion chromatography (SEC) (Fig 2A).

The final SEC profile revealed two peaks (Fig 2A). Specific binding of [^3^H]-DPN to KOR in eluted SEC fractions indicated that the second peak corresponded to active KOR reconstituted in Nanodiscs (Fig 2A). The Stokes diameter of Nanodiscs particles containing KOR was calculated to be close to 14.1 nm, which was slightly larger than the measured Stokes diameter of empty Nanodiscs, evaluated at 11.9 nm. Both values are in accordance with reported literature values of MSP1D1E3 Nanodiscs [15,45]. When fractions from the second peak were analyzed with SDS-PAGE (Fig 2B, S2 Fig), bands corresponding to both KOR (66 kDa) and the MSP1E3D1 protein around the Nanodiscs (32.6 kDa) were detected. In addition, a faint band just below 50 kDa was visible on the Western blot. This band may corresponds either to partially degraded receptors or non-glycosylated receptors as previously reported [50]. The presence of KOR was further verified both by western blotting (Fig 2B, S2 Fig) and MALDI mass spectrometry (S1 Table). The purity of isolated KOR/nanodics complexes was high when the Coomassie-stained gel was assessed and furthermore the intensity of the band corresponding to KOR was about half of the intensity of the band derived from the MSP protein (Fig 2B). This indicates the expected stoichiometry of one receptor for two MSP proteins, but it cannot be excluded that more than one receptor was reconstituted per Nanodisc. Taken together, the above characterization indicated that KOR was homogeneously isolated and reconstituted in Nanodiscs.

### Long-term stability of KOR in Nanodiscs

To perform functional studies of GPCRs in general, it is essential to stabilize and maintain the biological activity for a relatively long period of time. We used time-resolved SPA to investigate the evolution of high-affinity ligand binding to KOR reconstituted in Nanodiscs over time.

**Fig 3:**
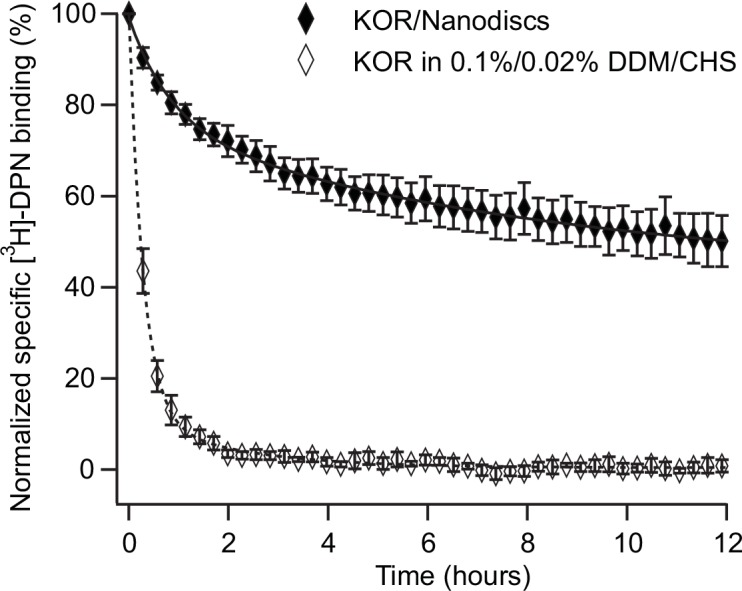
Time-resolved stability of KOR in detergent or when reconstituted in Nanodiscs. Stability of high-affinity ligand binding (10 nM [^3^H]-DPN ≈ 10 x *K*_*D*_) to KOR in 0.1%/0.02% DDM/CHS (w/v) micelles (open diamonds) or to KOR reconstituted in Nanodiscs (black diamonds) at 23°C. Specific [^3^H]-DPN binding was normalized to initial ligand binding at time zero where incubation at RT was initiated. Data are average of at least three experiments each performed in triplicates. Error bars represents standard error of the mean. Data were fitted to a double exponential decay curve to calculate the half-life of high-affinity ligand binding to KOR (see Table 1).

With only 30 fmol active receptors reconstituted in Nanodiscs, we were able to confirm the stability of high-affinity ligand binding over 12 hours at room temperature (Fig 3). KOR reconstituted in Nanodiscs showed a highly improved ability to bind [^3^H]-DPN with high-affinity for a prolong period of time compared to KOR in 0.1%/0.02% DDM/CHS. After counting for 100 minutes, where the ligand binding to KOR in detergent micelles was lost, KOR reconstituted in Nanodiscs maintained ligand binding with 73.5 ± 2.5% (n = 5) relative to the initial activity.

**Table 1:** Quantification of high-affinity ligand binding to KOR in detergent micelles or when reconstituted in Nanodiscs.

	*N*_*1*_ (%)	*τ*_*1*_ (hours)	*t*_*1*_ (hours)	*N*_*2*_ (%)	*τ*_*2*_ (hours)	*t*_*2*_ (hours)	*T*_*average*_ (hours)
KOR in DDM micelles*	97.4 ± 1.5	0.4 ± 0.01	0.3 ± 0.01	-	-	-	-
KOR in Nanodiscs	29.4 ± 0.8	1.2 ± 0.1	0.8 ± 0.1	68.9 ± 0.7	36.8 ± 1.6	25.5 ± 1.1	17.8 ± 1.1

Data presented in Fig 3, were fitted to either a single- (*) or a double exponential decay function. Half-life (*t*) was calculated from the exponential time constant (*τ*). *N* denotes the initial active receptor population (t = 0) calculated from the fit. The average half-life (*T*_*average*_) was calculated from *N*_*1*_, *N*_*2*_, *t*_*1*_ and *t*_*2*_. Errors represent standard deviation from the fit.

As for KOR in DDM micelles, the time-dependent traces of ligand-binding activity were fitted to an exponential decay function to quantify the half-life of receptor activity. However, two exponentials were necessary to fit the data of KOR reconstituted in Nanodiscs properly, which suggested that at least two populations of receptors were present in the sample. As indicated by the SEC profile (Fig 2), a part of the receptor/Nanodisc complexes were aggregated and present in the Superdex200 void volume. However, this fraction may correspond to the component of receptor with reduced stability.

Analyzed from the fitted curve, the stability was improved approximately 70 fold when the average half-life (*T*_*average*_, 17.8 ± 1.1 hours) of ligand binding activity to KOR reconstituted in Nanodiscs was compared to the half-life (0.25 ± 0.01 hours) obtained for KOR in DDM micelles (Fig 3 and Table 1). For the major part of the receptor population reconstituted in Nanodiscs (68.9%) the half-life of the slow component of ligand binding decay was 25.5 ± 1.1 hours. The improved stability of the majority of reconstituted KOR facilitated the use of the receptor for further characterization.

### Pharmacological properties of KOR/Nanodisc complexes

**Fig 4:**
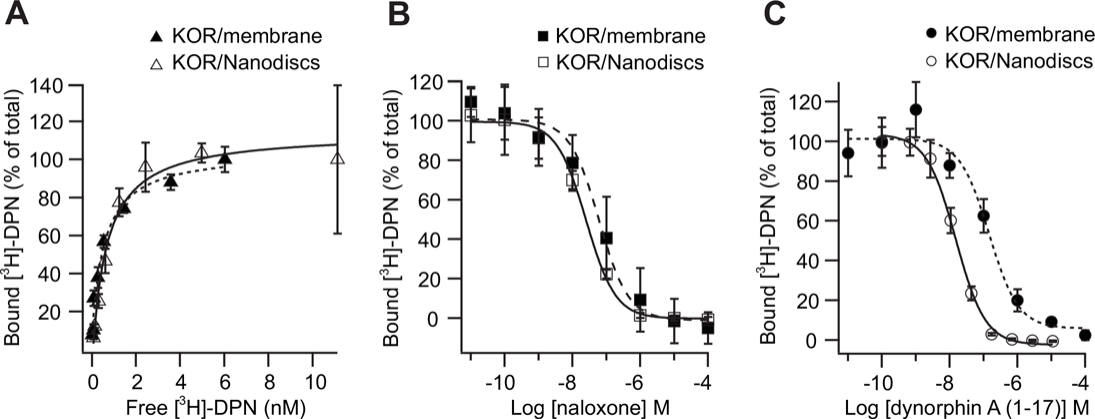
Pharmacology of KOR in isolated *P*. *pastoris* membrane and when reconstituted in Nanodiscs. A) Saturation binding assay of [^3^H]-DPN binding to KOR in membrane (black triangles) or KOR reconstituted in Nanodiscs (open triangles). From the specific binding the affinity constant, *K*_*D*_, of [^3^H]-DPN binding to KOR was calculated by fitting to a one-site binding model (see Table 2) B) Competition binding experiments with KOR antagonist naloxone and C) KOR agonist dynorphin A (1–17). Increasing concentrations of ligand competed the binding of 0.8 nM [^3^H]-DPN binding to KOR in *P*. *pastoris* cell membrane (black marks) or reconstituted in Nanodiscs (open marks). The calculated binding constants (*K*_*i*_) are shown in Table 2. Data shown are representative experiments of specific binding normalized to initial binding of [^3^H]-DPN binding to KOR. Data points are shown with standard deviation of triplicate measurements in one assay. All binding experiments were repeated at least three times.

To confirm that the receptor function was preserved during isolation and reconstitution into Nanodiscs, the pharmacology of KOR was characterized by applying SPA as a classical activity assay (Fig 4).

The affinity constant (*K*_*D*_) of [^3^H]-DPN binding to KOR/Nanodisc complexes was calculated to 0.84 ± 0.15 nM, comparable to the affinity of membrane bound receptors calculated to 0.94 ± 0.14 nM (Fig 4A, Table 2) and in agreement with literature values [32,51] (Table 2). To further investigate the pharmacology of KOR in *P*. *pastoris* cell membrane and when reconstituted in Nanodiscs, the antagonist naloxone and the peptide agonist dynorphin A (1–17) were tested in competition binding experiments using SPA. Calculated *IC*_*50*_ values were converted into inhibition constants (*K*_*i*_*)* and are listed in Table 2. Inhibition of [^3^H]-DPN with unlabeled naloxone showed similar pharmaceutical behavior of KOR in Nanodiscs and in membranes (Table 2 and Fig 4B). In contrast, a significant shift in *K*_*i*_ was measured for dynorphin A (1–17), indicating a higher affinity for the peptide to the receptor when reconstituted in Nanodiscs compared to KOR in *P*. *pastoris* membrane (Table 2 and Fig 4C). Additionally, the measured affinity of dynorphin A (1–17) for KOR in Nanodiscs is in accordance with literature values and indicates that the lipid composition can be important for high affinity binding of dynorphin A (1–17) to KOR.

**Table 2:** Comparison of binding affinities (*K*_*D*_, *K*_*i*_) of KOR in *P*. *pastoris* membranes or in Nanodiscs.

	*P*.*Pastoris* membrane	KOR/Nanodiscs	Literature
[3H]-DPN, *K*_*D*_	0.9 ± 0.1	0.8 ± 0.2	0.8
Naloxone, *K*_*i*_	36.3 ± 12.8	16.7 ± 2.9	9.6
Dynorphin A (1–17), *K*_*i*_	104.8 ± 57.2	6.1 ± 2.1	0.4–14.9

Calculated affinity constants (*K*_*D*_, nM) and displacement constants (*K*_*i*_, nM) of ligand binding to KOR in *P*. *pastoris* membranes or to KOR/Nanodisc complexes. Literature values are from [31,52].

**Fig 5:**
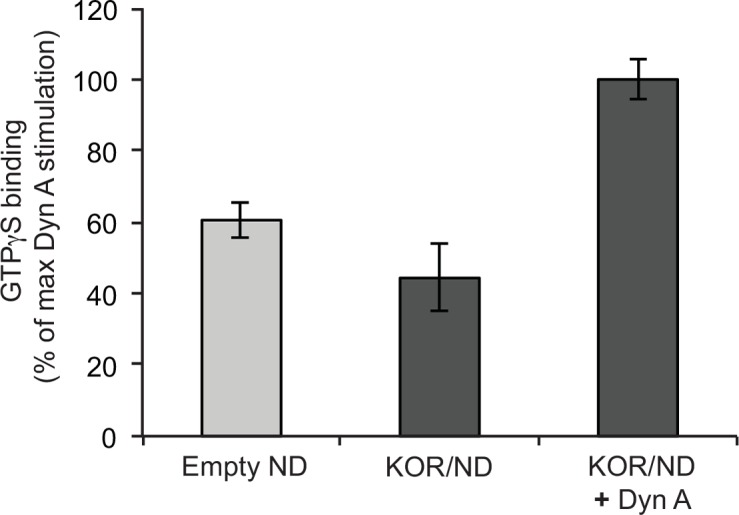
Isolated KOR reconstituted in Nanodiscs activated G_i_ protein. BODIPY FL GTPγS was used to confirm Gα_i_ protein activation by KOR after isolation and reconstitution into Nanodiscs. The intrinsic activity of G proteins was measured in the presence of empty Nanodiscs (light grey, Empty ND). Compared to KOR/Nanodisc sample without agonist stimulation (KOR/ND, dark grey), the fluorescence from BODIPY FL GTPγS binding to Gα_i_ increased by 56% when the KOR/Nanodisc sample was stimulated with 1 μM dynorphin A (1–17) (KOR/ND + Dyn A, dark grey). Error bars represent standard error of the mean between two independent experiments.

KOR activation is furthermore defined by agonist-induced binding of the Gα-subunit of the heterotrimeric G proteins of the G_i/o_ family. In addition, some studies indicate that KOR can also couple to G_q/11_ proteins and activate the phospholipase C pathway [53]. To confirm that the isolated and reconstituted KOR was able to activate G-proteins, we used the non-hydrolysable fluorescently labeled BODIPY FL GTPγS to measure receptor-catalyzed GTPγS binding to purified Gα_i_ in the presence of Gβγ (Fig 5). Due to a change in the chemical environment near the fluorophore, the fluorescence of BODIPY FL GTPγS increases when it binds to the Gα subunit [35]. When KOR was stimulated by dynorphin A (1–17), a substantial increase in the fluorescence signal from BODIPY FL GTPγS was detected, when compared to both the signal from the assay performed in the absence of ligand and from the assay done with empty Nanodiscs (Fig 5). Compared to KOR/Nanodisc samples in the absence of agonist, the fluorescence of agonist stimulated KOR/Nanodisc sample increased by 56% (Fig 5). This preliminary data suggest that KOR reconstituted in Nanodiscs can activate G_i_ proteins in vitro.

The binding of different ligands to KOR and receptor-stimulated G-protein activation, suggest that KOR was successfully reconstituted in Nanodiscs and maintained a pharmacology that was comparable or even improved compared to KOR in *P*. *pastoris* membrane.

## Discussion

A major bottleneck, for MP investigation and the use of isolated MPs in early stage of drug discovery, is that MPs need to be extracted from the membrane using detergents, which often destabilize their biological function. Furthermore, MPs remain in detergent solution through often extensive purification protocols and during subsequent experiments. This is for instance the case when purified MPs are immobilized on a biosensor surface for screening of fragment libraries and for measuring the kinetics of ligand binding, which can last for several hours [19,54,55]. Consequently long-term functional stability of MPs in solution is a key requirement, in order to conduct investigations at the molecular level.

The stability of MPs in detergent solution can be measured by several different analytical techniques. The most common method is to measure the thermostability of proteins by circular dichroism spectroscopy, differential scanning calorimetry or binding of fluorescent dyes to exposed hydrophobic residues. These techniques are limited, both because they require purified sample and often relatively large quantities. Many MPs, especially GPCRs, are not sufficiently stable in detergent solution to be purified to a reasonable level and in sufficient quantities. Furthermore, the methods do not determine if receptors retain their native pharmacology [37,56]. Here, we used time-resolved SPA as an assay to quantify high-affinity ligand binding to a pharmaceutical relevant GPCR in detergent micelles, and to validate long-term stabilization of Nanodisc-reconstituted receptors. Compared to the above-mentioned techniques, the stability could be assayed directly after membrane solubilization without prior purification, since the receptor was immobilized on SPA beads from crude detergent extracted membrane fragments. Moreover, thermal stability assays would be inappropriate to assess Nanodisc reconstituted receptor stability, since they rely on thermal denaturation curves, where the whole lipoprotein complex would interfere with the measurements.

Compared to conventional radioligand binding assays that rely on separation techniques, such as filter binding assays, SPA binding assay avoid usual filtration and washing procedures. Standard filtration assays can be challenging for dissolved MPs or nanoparticles in general, because of the relatively large filter pore size and thus loss of sample. Furthermore, the obtained information about a given receptor activity is limited to single endpoints at a given time. Using SPA as a time-resolved assay, where the same assay was counted repeatedly, the obtained data had a high degree of information. From the time-resolved data, the receptor activity monitored over a prolonged period of time could be fitted to an exponential decay function and the half-life of high-affinity ligand binding could be quantified. In addition, for a complete time-resolved depiction of receptor activity, the consumption of receptor sample (30 fmol) and assay reagents were minimal. Since SPA is a homogeneous”mix-and-measure assay” several conditions could be tested in parallel, and the assay could easily be automated to assess receptor activity over time directly.

We investigated the long-term functional stability of isolated KOR, in detergent and after reconstitution into Nanodiscs, using SPA as a time-resolved assay to measure the decay of high-affinity ligand binding. The human KOR was overexpressed in *P*. *pastoris* and extracted from the cell membrane by DDM, a detergent that is often successfully used for GPCRs solubilization. When SPA was used to analyze the time course of [^3^H]-DPN binding to KOR at room temperature, the signal decreased rapidly with a calculated half-life of 15.3 ± 0.5 minutes and undetectable after 100 minutes. The same fast decrease in binding was measured with a different separation technique, which confirmed that SPA is valid to assay ligand-binding stability over time (Fig 1B).

Because of the unstable nature of KOR in DDM solution, extracted receptors were not suitable for further investigation, even though DDM has proven to be a suitable detergent for KOR solubilization [31,41]. The expression system used in this study, may affect the stability of solubilized receptors. Furthermore KOR used for structural studies, was genetically modified to improve the thermal stability of the protein [31]. The functional long-term stability of KOR reconstituted in Nanodiscs was remarkably increased, compared to receptors in detergent solution (Fig 3, Table 1). When analyzed with time-resolved SPA the half-life of the high-affinity ligand binding to KOR, was improved from 15.3 ± 0.5 minutes to 17.8 ± 1.1 hours and the receptor was active for up to 12 hours at room temperature. To our knowledge, only one study investigated the biological stability of a GPCR over time when reconstituted in rHDL particles, comparable to Nanodiscs. The time dependent binding of conformational sensitive antibodies to a GPCR was followed by immobilization on a SPR sensor chip. In DDM micelles, the binding of antibodies decreased by 93% within 24 hours at 4°C, whereas only 15% antibody binding was lost when the receptor was reconstituted in rHDL [10]. The functional stability of KOR reconstituted in Nanodiscs is comparable and thus meets the requirements of long-term stability to perform measurements with e.g. SPR.

After reconstitution of KOR in Nanodiscs, the pharmacology of KOR was preserved and comparable to KOR embedded in *P*. *pastoris* membrane or even improved in the case of the peptide agonist dynorphin A (1–17) (Fig 4). Compared to KOR in *P*. *pastoris* membrane patches, the affinity of dynorphin A (*K*_*i*_) was enriched 20-fold when receptors were reconstituted in Nanodiscs. The increased affinity indicates that the lipid composition can be of importance for the interaction between KOR and the peptide agonist dynorphin A (1–17) [57,58].

In addition, G protein activation by KOR was confirmed by increased binding level of BODIPY FL GTPγS to Gα_i_ when KOR was stimulated by dynorphin A (1–17) (Fig 4). This is the first time G protein activation is demonstrated for KOR *in vitro*. Such assay format is important to demonstrate the actual activity of a GPCR and to characterize the intrinsic nature of ligands acting as agonists, antagonists or inverse agonists. Furthermore, isolated KOR can be used to investigate the molecular interactions controlling activation of different G protein subtype pathways or for investigation of biased agonism, where ligands selectively induce either G protein or β-arrestin-dependent signaling pathways. For these *in vitro* investigations a functional receptor stable for longer periods is crucial.

## Conclusion

Long-term stability of intrinsic unstable MPs is the key to obtain high quality data to understand MPs molecular mechanisms and to develop new highly selective drug-candidates. We have shown that time-resolved SPA has the potential to efficiently screen and identify conditions that can improve functional stability of a highly fragile GPCR. SPA counted repeatedly was used as a straightforward "mix-and-read" assay, to access information on long-term stability of high-affinity ligand binding to KOR. Using this approach, we showed how the functional long-term stability of KOR was highly improved from being in detergent solution and after a successful reconstitution into Nanodiscs. The functional stability of receptors was necessary to characterize the pharmacology of KOR reconstituted in Nanodiscs.

SPA is already a common technique, used in both academia and in the pharmaceutical industry. The variety of beads and immobilization strategies available, such as beads functionalized with nickel chelate to immobilize proteins with a histidine affinity tag, makes the presented method relevant for a wide range of MPs. Time-resolved SPA can be used to optimize the conditions for long-term stability of MPs, that is required for many techniques used to understand MPs at the molecular level and to develop new drug candidates from compound screenings with isolated proteins as targets.

## Supporting Information

S1 Fig Stability of high-affinity ligand binding to KOR solubilized in various detergents measured by time-resolved SPA. Several detergents was tested, here an example of 1%/0.2% DDM compared to 0.5%/0.1% CHAPS is shown. *P*. *Pastoris* membranes were solubilized in either 0.5%/0.1% CHAPS/CHS (red) or 1%/0.2% DDM/CHS (blue). To measure specific binding of radioligand to KOR, solubilized proteins were mixed with streptavidin SPA beads and [^3^H]-DPN at saturating concentration for high-affinity ligand binding (10 nM [^3^H]-DPN ≈ 10x*K*_*D*_). Non-specific binding was measured in parallel by including 50 μM naloxone. The final concentration of detergents after sample dilution in the assay was; 0.1%/0.02% for DDM/CHS and 0.5/0.2% for CHAPS/CHS. The assay was repeatedly counted at room temperature. Specific [^3^H]-DPN binding to KOR was normalized to initial activity of each sample. The observed decay of radioligand binding to KOR could be fitted to a first-order exponential decay function (dashed lines). The half-life of high-affinity ligand binding to KOR in DDM micelles was 15.3 ± 0.5 minutes and for KOR in CHAPS micelles the half-life was measured to 69.3 ± 2.8 minutes. The quantity of total solubilized protein in CHAPS detergent (10.3 ± 0.3%) was lower than for DDM solubilization (57.1% ± 3.6%). Therefore the later was used for further purification even though the stability of ligand binding was lower. All data points are average of three independent experiments each performed in triplicates. Error bars represent the standard error of the mean.
(DOCX)

S2 Fig SDS-PAGE detection of KOR, uncropped and unadjusted gel lanes. A) Coomassie brilliant blue staining. B) Western blot detection with streptavidin alkaline phosphatase binding to the C-terminal biotin-tag of KOR.
(DOCX)

S1 Table Identification of KOR by MALDI mass spectrometry. Sequence coverage was 7% and the protein was identified as kappa-type opioid receptor isoform 2 (Homo sapiens), which suggest that the identified protein was KOR.
(DOCX)

## Abbreviations:

CHS: Cholesteryl hemisuccinate
DDM: n-Dodecyl-β-D-maltoside
DPN: Diprenorpine
Dyn A: Dynorphin A
GPCRs: G protein coupled receptors
IMAC: Immobilized metal ion affinity chromatography
KOR: Kappa opioid receptor
MPs: Membrane proteins
MSPs: Membrane scaffold proteins
NMR: Nuclear magnetic resonance
POPC: 1-palmitoyl-2-oleoyl-*sn*-glycero-3-phosphocholine
POPG: 1-palmitoyl-2-oleoyl-*sn*-glycero-3-phospho-(1'-*rac*-glycerol)
P. pastoris: Pichia pastoris
rHDL: High-density lipoprotein phospholipid bilayer particle
SEC: Size exclusion chromatography
Sf9: Spodoptera frugiperda
SPA: Scintillation proximity assay
SPR: Surface plasmon resonance
TEV: Tobacco etch virus
T4L: T4 lysozyme

## References

[1] Yıldırım MA, Goh K-I, Cusick ME, Barabási A-L, Vidal M. Drug—target network. Nat Biotechnol. 2007;25: 1119–1126. 17921997 10.1038/nbt1338

[2] Alkhalfioui F, Magnin T, Wagner R. From purified GPCRs to drug discovery: the promise of protein-based methodologies. Curr Opin Pharmacol. 2009;9: 629–635. 10.1016/j.coph.2009.04.002 19443270

[3] Mayr LM, Fuerst P. The future of high-throughput screening. J Biomol Screen. 2008;13: 443–448. 10.1177/1087057108319644 18660458

[4] Lindert S, Maslennikov I, Chiu EJC, Pierce LC, McCammon JA, Choe S. Drug screening strategy for human membrane proteins: from NMR protein backbone structure to in silica- and NMR-screened hits. Biochem Biophys Res Commun. 2014;445: 724–733. 10.1016/j.bbrc.2014.01.179 24525125 PMC4457379

[5] Chen D, Errey JC, Heitman LH, Marshall FH, IJzerman AP, Siegal G. Fragment Screening of GPCRs Using Biophysical Methods: Identification of Ligands of the Adenosine A2A Receptor with Novel Biological Activity. ACS Chem Biol. 2012;7: 2064–2073. 10.1021/cb300436c 23013674

[6] Glickman JF, Schmid A, Ferrand S. Scintillation Proximity Assays in High-Throughput Screening. Assay Drug Dev Technol. 2008;6: 433–455. 10.1089/adt.2008.135 18593378

[7] Lea WA, Simeonov A. Fluorescence polarization assays in small molecule screening. Expert Opin Drug Discov. 2011;6: 17–32. 10.1517/17460441.2011.537322 22328899 PMC3277431

[8] Fang Y. Ligand-receptor interaction platforms and their applications for drug discovery. Expert Opin Drug Discov. 2012;7: 969–988. 22860803 10.1517/17460441.2012.715631

[9] Hagn F, Etzkorn M, Raschle T, Wagner G. Optimized Phospholipid Bilayer Nanodiscs Facilitate High-Resolution Structure Determination of Membrane Proteins. J Am Chem Soc. 2013;135: 1919–25. 10.1021/ja310901f 23294159 PMC3566289

[10] Yoshiura C, Kofuku Y, Ueda T, Mase Y, Yokogawa M, Osawa M, et al NMR Analyses of the Interaction between CCR5 and Its Ligand Using Functional Reconstitution of CCR5 in Lipid Bilayers. J Am Chem Soc. 2010;132: 6768–6777. 10.1021/ja100830f 20423099

[11] Patching SG. Surface plasmon resonance spectroscopy for characterisation of membrane protein-ligand interactions and its potential for drug discovery. Biochim Biophys Acta. 2014;1838: 43–55. 10.1016/j.bbamem.2013.04.028 23665295

[12] Locatelli-Hoops S, Yeliseev AA, Gawrisch K, Gorshkova I. Surface plasmon resonance applied to G protein-coupled receptors. Biomed Spectrosc Imaging. 2013;2: 155–181. 24466506 10.3233/BSI-130045 PMC3898597

[13] Jamshad M, Charlton J, Lin Y, Routledge SJ, Bawa Z, Knowles TJ, et al G-protein coupled receptor solubilization and purification for biophysical analysis and functional studies, in the total absence of detergent. Biosci Rep. 2015;35: 1–10. 10.1042/BSR20140171 PMC4400634 25720391

[14] Tribet C, Diab C, Dahmane T, Zoonens M, Popot J, Winnik F. Thermodynamic Characterization of the Exchange of Detergents and Amphipols at the Surfaces of Integral Membrane Proteins. Langmuir. 2009;25: 12623–12634. 10.1021/la9018772 19594168

[15] Bayburt TH, Sligar SG. Membrane protein assembly into Nanodiscs. FEBS Lett. 2010;584: 1721–1727. 10.1016/j.febslet.2009.10.024 19836392 PMC4758813

[16] Tate CG, Schertler GFX. Engineering G protein-coupled receptors to facilitate their structure determination. Curr Opin Struct Biol. 2009;19: 386–395. 10.1016/j.sbi.2009.07.004 19682887

[17] Zoonens M, Popot J-L. Amphipols for each season. J Membr Biol. 2014;247: 759–796. 10.1007/s00232-014-9666-8 24969706 PMC4282167

[18] Chae P, Rasmussen SG, Rana R, Gotfryd K, Chandra R, Goren M, et al Maltose-neopentyl glycol (MNG) amphiphiles for solubilization, stabilization and crystallization of membrane proteins. Nat Methods. 2010;7: 1003–1008. 10.1038/nmeth.1526 21037590 PMC3063152

[19] Christopher JA, Brown J, Dore AS, Errey JC, Koglin M, Marshall FH, et al Biophysical Fragment Screening of the β1-Adrenergic Receptor: Identification of High Affinity Arylpiperazine Leads Using Structure-Based Drug Design. J Med Chem. 2013;56: 3446–3455. 10.1021/jm400140q 23517028 PMC3654563

[20] Andrews SP, Brown GA, Christopher JA. Structure-based and fragment-based GPCR drug discovery. ChemMedChem. 2014;9: 256–275. 10.1002/cmdc.201300382 24353016

[21] Rich RL, Errey J, Marshall F, Myszka DG. Biacore analysis with stabilized G-protein-coupled receptors. Anal Biochem. 2011;409: 267–272. 10.1016/j.ab.2010.10.008 20969829 PMC3010267

[22] Das A, Zhao J, Schatz GC, Sligar S, Van Duyne RP. Screening of type I and II drug binding to human cytochrome P450-3A4 in nanodiscs by localized surface plasmon resonance spectroscopy. Anal Chem. 2009;81: 3754–3759. 10.1021/ac802612z 19364136 PMC4757437

[23] Borch J, Torta F, Sligar SG, Roepstorff P. Nanodiscs for immobilization of lipid bilayers and membrane receptors: kinetic analysis of cholera toxin binding to a glycolipid receptor. Anal Chem. 2008;80: 6245–6252. 10.1021/ac8000644 18616345

[24] Fox DA, Larsson P, Lo RH, Kroncke BM, Kasson PM, Columbus L. Structure of the Neisserial Outer Membrane Protein Opa60: Loop Flexibility Essential to Receptor Recognition and Bacterial Engulfment. J Am Chem Soc. 2014;136: 9938–9946. 10.1021/ja503093y 24813921 PMC4105060

[25] Quick M, Javitch J. Monitoring the function of membrane transport proteins in detergent-solubilized form. Proc Natl Acad Sci U S A. 2007;104: 3603–3608. 17360689 10.1073/pnas.0609573104 PMC1805550

[26] De Jong L, Uges D, Franke J, Bischoff R. Receptor–ligand binding assays: technologies and applications. J Chromatogr B Anal Technol Biomed Life Sci. 2005;829: 1–25. 10.1016/j.jchromb.2005.10.002 16253574

[27] Nasr ML, Singh SK. Radioligand Binding to Nanodisc-Reconstituted Membrane Transporters Assessed by the Scintillation Proximity Assay. Biochemistry. 2014;53: 4–6. 10.1021/bi401412e 24344975 PMC4062192

[28] Fiez-Vandal C, Leder L, Freuler F, Sykes D, Charlton S, Siehler S, et al HDL-like discs for assaying membrane proteins in drug discovery. Biophys Chem. 2012;165–166: 56–61. 10.1016/j.bpc.2012.03.005 22542136

[29] Butelman ER, Yuferov V, Kreek MJ. κ-opioid receptor/dynorphin system: genetic and pharmacotherapeutic implications for addiction. Trends Neurosci. 2012;35: 587–596. 10.1016/j.tins.2012.05.005 22709632 PMC3685470

[30] Schwarzer C. 30 years of dynorphins—New insights on their functions in Neuropsychiatric diseases. Pharmacol Ther. 2009;123: 353–370. 10.1016/j.pharmthera.2009.05.006 19481570 PMC2872771

[31] Wu H, Wacker D, Mileni M, Katritch V, Han GW, Vardy E, et al Structure of the human κ-opioid receptor in complex with JDTic. Nature. 2012;485: 327–332. 10.1038/nature10939 22437504 PMC3356457

[32] André N, Cherouati N, Prual C, Steffan T, Zeder-Lutz G, Magnin T, et al Enhancing functional production of G protein-coupled receptors in *Pichia pastoris* to levels required for structural studies via a single expression screen. Protein Sci. 2006;15: 1115–1126. f 16597836 10.1110/ps.062098206 PMC2242496

[33] Ritchie T, Grinkova Y, Bayburt T, Denisov I, Zolnerciks J, Atkins W, et al Reconstitution of Membrane Proteins in Phospholipid Bilayer Nanodiscs. Methods Enzymol. 2009;464: 211–231. 10.1016/S0076-6879(09)64011-8 19903557 PMC4196316

[34] Rasmussen S, Devree B, Zou Y, Kruse A, Chung K, Kobilka T, et al Crystal structure of the β_2_ adrenergic receptor–G_s_ protein complex. Nature. 2011; 549–555. 10.1038/nature10361 PMC3184188 21772288

[35] Mcewen D, Gee K, Kang H, Neubig R. Fluorescent BODIPY-GTP Analogs: Real-Time Measurement of Nucleotide Binding to G Proteins. Anal Biochem. 2001;291: 109–117. 11262163 10.1006/abio.2001.5011

[36] Yao Z, Kobilka B. Using synthetic lipids to stabilize purified β_2_ adrenoceptor in detergent micelles. Anal Biochem. 2005;343: 344–346. 16005425 10.1016/j.ab.2005.05.002

[37] Cooke R, Koglin M, Errey J, Marshall F. Preparation of purified GPCRs for structural studies. Biochem Soc Trans. 2013;41: 185–190. 10.1042/BST20120240 23356281

[38] Bosworth N, Towers P. Scintillation proximity assay. Nature. 1989;341: 167–168. 2550824 10.1038/341167a0

[39] Kuszak AJ, Pitchiaya S, Anand JP, Mosberg HI, Walter NG, Sunahara RK. Purification and functional reconstitution of monomeric μ-opioid receptors: allosteric modulation of agonist binding by G_i2_. J Biol Chem. 2009;284: 26732–26741. 10.1074/jbc.M109.026922 19542234 PMC2785361

[40] Shibata Y, Gvozdenovic-Jeremic J, Love J, Kloss B, White JF, Grisshammer R, et al Optimising the combination of thermostabilising mutations in the neurotensin receptor for structure determination. Biochim Biophys Acta. 2013;1828: 1293–1301. 10.1016/j.bbamem.2013.01.008 23337476 PMC3582860

[41] Howell SC, Mittal R, Huang L, Travis B, Breyer RM, Sanders CR. CHOBIMALT: A Cholesterol-Based Detergent. Biochemistry. 2010;49: 9572–9583. 10.1021/bi101334j 20919740 PMC3030671

[42] D’Antona AM, Xie G, Sligar S, Oprian DD. Assembly of an Activated Rhodopsin−Transducin Complex in Nanoscale Lipid Bilayers. Biochemistry. 2014;53: 127–134. 10.1021/bi4012995 24328127 PMC3939051

[43] Civjan NR, Bayburt TH, Schuler MA, Sligar SG. Direct solubilization of heterologously expressed membrane proteins by incorporation into nanoscale lipid bilayers. Biotechniques. 2003;35: 556–563. 14513561 10.2144/03353rr02

[44] Marty MT, Wilcox KC, Klein WL, Sligar SG. Nanodisc-solubilized membrane protein library reflects the membrane proteome. Anal Bioanal Chem. 2013;405: 4009–4016. 10.1007/s00216-013-6790-8 23400332 PMC3628400

[45] Mitra N, Liu Y, Liu J, Serebryany E, Mooney V, DeVree BT, et al Calcium-dependent ligand binding and G-protein signaling of family B GPCR parathyroid hormone 1 receptor purified in nanodiscs. ACS Chem Biol. 2013;8: 617–625. 10.1021/cb300466n 23237450 PMC4015634

[46] Bayburt T, Vishnivetskiy S, Mclean M, Morizumi T, Huang C, Tesmer J, et al Monomeric Rhodopsin Is Sufficient for Normal Rhodopsin Kinase (GRK1) Phosphorylation and Arrestin-1 Binding. J Biol Chem. 2011;286: 1420–1428. 10.1074/jbc.M110.151043 20966068 PMC3020750

[47] Bayburt T, Leitz A, Xie G, Oprian D, Sligar S. Transducin Activation by Nanoscale Lipid Bilayers Containing One and Two Rhodopsins. J Biol Chem. 2007;282: 14875–14881. 17395586 10.1074/jbc.M701433200

[48] Whorton MR, Bokoch MP, Rasmussen SG, Huang B, Zare RN, Kobilka B, et al A monomeric G protein-coupled receptor isolated in a high-density lipoprotein particle efficiently activates its G protein. Proc Natl Acad Sci U S A. 2007;104: 7682–7687. 17452637 10.1073/pnas.0611448104 PMC1863461

[49] Whorton MR, Jastrzebska B, Park PS, Fotiadis D, Engel A, Palczewski K, et al Efficient coupling of transducin to monomeric rhodopsin in a phospholipid bilayer. J Biol Chem. 2008;283: 4387–4394. 18033822 10.1074/jbc.M703346200 PMC2651572

[50] Magnin T, Fiez-Vandal C, Potier N, Coquard A, Leray I, Steffan T, et al A novel, generic and effective method for the rapid purification of G protein-coupled receptors. Protein Expr Purif. 2009;64: 1–7. 10.1016/j.pep.2008.09.007 18835448

[51] Xu W, Chen C, Huang P, Li J, Riel JK De, Javitch JA. The Conserved Cysteine 7.38 Residue Is Differentially Accessible in the Binding-Site Crevices of the μ, δ, and κ Opioid Receptors. Biochemistry. 2000;39: 13904–13915. 11076532 10.1021/bi001099p

[52] Bradford CS, Walthers E a, Searcy BT, Moore FL. Cloning, heterologous expression and pharmacological characterization of a kappa opioid receptor from the brain of the rough-skinned newt, Taricha granulosa. J Mol Endocrinol. 2005;34: 809–23. 15956349 10.1677/jme.1.01711

[53] Bruchas M, Chavkin C. Kinase cascades and ligand-directed signaling at the kappa opioid receptor. Psychopharmacology (Berl). 2010;210: 137–147. 20401607 10.1007/s00213-010-1806-y PMC3671863

[54] Aristotelous T, Ahn S, Shukla AK, Gawron S, Sassano MF, Kahsai AW, et al Discovery of β2 Adrenergic Receptor Ligands Using Biosensor Fragment Screening of Tagged Wild-Type Receptor. ACS Med Chem Lett. 2013;4: 1005–1010. 24454993 10.1021/ml400312j PMC3892729

[55] Shepherd CA, Hopkins AL, Navratilova I. Fragment screening by SPR and advanced application to GPCRs. Prog Biophys Mol Biol. 2014;116: 113–123. 10.1016/j.pbiomolbio.2014.09.008 25301577

[56] Alexandrov AI, Mileni M, Chien EYT, Hanson M a, Stevens RC. Microscale fluorescent thermal stability assay for membrane proteins. Structure. 2008;16: 351–9. 10.1016/j.str.2008.02.004 18334210

[57] Sargent DF, Schwyzer R. Membrane lipid phase as catalyst for peptidereceptor interactions. Proc Natl Acad Sci USA. 1986;83: 5774–5778. 2874556 10.1073/pnas.83.16.5774 PMC386377

[58] Björnerås J, Gräslund A, Mäler L. Membrane interaction of disease-related dynorphin A variants. Biochemistry. 2013;52: 4157–4167. 10.1021/bi4004205 23705820

