# Supplementary material for: Functional Stability of the Human Kappa Opioid Receptor Reconstituted in Nanodiscs Revealed by a Time-Resolved Scintillation Proximity Assay
Source: PLoS One. 2016 Apr 1;11(4):e0150658. doi: 10.1371/journal.pone.0150658 (PMC4817975; doi:10.1371/journal.pone.0150658)
Supplement: S1 Fig — Several detergents was tested, here an example of 1%/0.2% DDM compared to 0.5%/0.1% CHAPS is shown. P. Pastoris membranes were solubilized in either 0.5%/0.1% CHAPS/CHS (red) or 1%/0.2% DDM/CHS (blue). To measure specific binding of radioligand to KOR, solubilized proteins were mixed with streptavidin SPA beads and [3H]-DPN at saturating concentration for high-affinity ligand binding (10 nM [3H]-DPN ≈ 10xKD). Non-specific binding was measured in parallel by including 50 μM naloxone. The final concentration of detergents after sample dilution in the assay was; 0.1%/0.02% for DDM/CHS and 0.5/0.2% for CHAPS/CHS. The assay was repeatedly counted at room temperature. Specific [3H]-DPN binding to KOR was normalized to initial activity of each sample. The observed decay of radioligand binding to KOR could be fitted to a first-order exponential decay function (dashed lines). The half-life of high-affinity ligand binding to KOR in DDM micelles was 15.3 ± 0.5 minutes and for KOR in CHAPS micelles the half-life was measured to 69.3 ± 2.8 minutes. The quantity of total solubilized protein in CHAPS detergent (10.3 ± 0.3%) was lower than for DDM solubilization (57.1% ± 3.6%). Therefore the later was used for further purification even though the stability of ligand binding was lower. All data points are average of three independent experiments each performed in triplicates. Error bars represent the standard error of the mean. (DOCX) [file pone.0150658.s001.docx]

# Supporting Information

**S1 Fig. Stability of high-affinity ligand binding to KOR solubilized in various detergents measured by time-resolved SPA.** Several detergents was tested, here an example of 1%/0.2% DDM compared to 0.5%/0.1% CHAPS is shown. *P. Pastoris* membranes were solubilized in either 0.5%/0.1% CHAPS/CHS (red) or 1%/0.2% DDM/CHS (blue). To measure specific binding of radioligand to KOR, solubilized proteins were mixed with streptavidin SPA beads and [^3^H]-DPN at saturating concentration for high-affinity ligand binding (10 nM [^3^H]-DPN ≈ 10x*K_D_*). Non-specific binding was measured in parallel by including 50 μM naloxone. The final concentration of detergents after sample dilution in the assay was; 0.1%/0.02% for DDM/CHS and 0.5/0.2% for CHAPS/CHS. The assay was repeatedly counted at room temperature. Specific [^3^H]-DPN binding to KOR was normalized to initial activity of each sample. The observed decay of radioligand binding to KOR could be fitted to a first-order exponential decay function (dashed lines). The half-life of high-affinity ligand binding to KOR in DDM micelles was 15.3 ± 0.5 minutes and for KOR in CHAPS micelles the half-life was measured to 69.3 ± 2.8 minutes. The quantity of total solubilized protein in CHAPS detergent (10.3 ± 0.3 %) was lower than for DDM solubilization (57.1% ± 3.6 %). Therefore the later was used for further purification even though the stability of ligand binding was lower. All data points are average of three independent experiments each performed in triplicates. Error bars represent the standard error of the mean.
