# Supplementary material for: Functional Stability of the Human Kappa Opioid Receptor Reconstituted in Nanodiscs Revealed by a Time-Resolved Scintillation Proximity Assay
Source: PLoS One. 2016 Apr 1;11(4):e0150658. doi: 10.1371/journal.pone.0150658 (PMC4817975; doi:10.1371/journal.pone.0150658)
Supplement: S2 Fig — A) Coomassie brilliant blue staining. B) Western blot detection with streptavidin alkaline phosphatase binding to the C-terminal biotin-tag of KOR. (DOCX) [file pone.0150658.s002.docx]

# Supporting Information

**S2 Fig. SDS-PAGE detection of KOR, uncropped and unadjusted gel lanes.** A) Coomassie brilliant blue staining. B) Western blot detection with streptavidin alkaline phosphatase binding to the C-terminal biotin-tag of KOR.
