# Supplementary material for: Functional Stability of the Human Kappa Opioid Receptor Reconstituted in Nanodiscs Revealed by a Time-Resolved Scintillation Proximity Assay
Source: PLoS One. 2016 Apr 1;11(4):e0150658. doi: 10.1371/journal.pone.0150658 (PMC4817975; doi:10.1371/journal.pone.0150658)
Supplement: S1 Table — Sequence coverage was 7% and the protein was identified as kappa-type opioid receptor isoform 2 (Homo sapiens), which suggest that the identified protein was KOR. (DOCX) [file pone.0150658.s003.docx]

# Supporting Information

**S1 Table. Identification of KOR by MALDI mass spectrometry.**

| **Mass** | **Amino acid sequence** | **Amino acid number, location** | **Ions score** |
| --- | --- | --- | --- |
| 1388.64 | CFRDFCFPLK | 251-260 | 59 |
| 1175.59 | NTVQDPAYLR | 273-282 | 6 |

Sequence coverage was 7% and the protein was identified as kappa-type opioid receptor isoform 2 (Homo sapiens), which suggest that the identified protein was KOR.
